# Editorial: Digital health applications: acceptance, benefit assessment, and costs from the perspective of patients and medical professionals

**DOI:** 10.3389/frhs.2024.1501903

**Published:** 2024-10-30

**Authors:** Tonio Schoenfelder, Tom Schaal

**Affiliations:** ^1^ Department of Health Services Research, WIG2 GmbH, Leipzig, Germany; ^2^ Chair Health Sciences/Public Health, Medical Faculty, Technische Universität Dresden, Dresden, Germany; ^3^ Faculty of Health and Healthcare Sciences, University of Applied Sciences Zwickau, Zwickau, Germany

**Keywords:** digital health applications, digital health technology, health technology, technology acceptance, telemedicine, mHealth, eHealth, DiGA

**Editorial on the Research Topic**
Digital health applications: acceptance, benefit assessment, and costs from the perspective of patients and medical professionals

Digital health services have witnessed a growing prevalence in healthcare systems around the world, with diverse levels of implementation and hurdles to surmount. Germany took the lead in creating the possibility for prescribing digital health applications (DHAs) as CE-certified digital medical devices with costs covered by the national health system, a trend that has been emulated by other countries, such as France and Belgium (1). Nonetheless, digital healthcare services continue to be perceived as innovations in most healthcare systems, encountering challenges due to the absence of well-established reimbursement channels. In healthcare systems where statutory health insurance covers the costs of healthcare services, patients are often reluctant to pay for digital health services privately. Additionally, both patients and service providers lack initial experience with these innovations (2–4), giving rise to skepticism and requiring time for the innovation to integrate into healthcare.

To secure health insurance coverage for these services, clear evidence of patient benefits is generally required, involving substantial costs and time investment, which smaller innovative companies may find prohibitive. To expedite the integration of DHAs into healthcare systems, several countries have instituted measures to address these barriers. For instance, Germany has adopted a two-phase market entry process, in which an initial pilot study grants market access and reimbursement by the national health system, followed by a confirmatory study to definitively demonstrate the benefits (5). What started as an innovative tool to give patients quick access to DHAs, especially for psychological indications where therapist waiting times are often long, has unfortunately become a very complex system. The requirements for studies to prove a benefit for patients are now much higher, making the studies time consuming and very expensive, so that hardly any new DHA is made available (6).

In recent years, digital health services have gained substantial momentum, revolutionizing healthcare delivery and presenting new opportunities for patient care. In this research topic the contributing authors present a variety of perspectives on the digitalisation of the health care sector. For example, Giebel et al. developed the DiGA-Care Path, a step-by-step analysis of DHA supply in Germany. This approach comprises a “main path,” concentrating on the supply environment, and a “sub-path,” illustrating the supply delivered by the DHA. This methodology assists in identifying problems and potential quality improvements in the current DHA supply and can serve as a guidance for international policymakers and stakeholders.

Stapelfeldt et al. performed a systematic assessment of DHA intended to treat obesity (7). The study concluded that most apps partially meet guideline recommendations and exhibit adequate to good quality based on the MARS score. However, evaluating the quality of mobile health applications remains challenging for patients, despite their low-threshold accessibility.

Naemi et al. designed and evaluated an Electronic Health Record (EHR) for amblyopia patients in Iran, aiming to enhance information management and reduce treatment costs. A usability evaluation showed that over 90% of users rated the web-based EHR system as very good or good, demonstrating high patient acceptance. Implementing an EHR for amblyopia has the potential to improve care quality and facilitate complication control.

Tischendorf et al. examined the sustainable integration of digitalization in nursing education, pinpointing trends in digitalization-related training and emphasizing the importance of involving nursing professionals in digital technology development. The literature review suggests that discussions on this topic in German-language literature lag behind those in the international context, highlighting the need for collaboration between nursing professionals and nursing sciences.

Arabian et al. investigated patients’ understanding of electronic prescriptions in Iran, underscoring the necessity for improved patient comprehension of the potential consequences of such technology on their relationships with healthcare providers. Active patient engagement and positive attitudes toward electronic prescription systems can enhance healthcare service quality, increase acceptance, and simplify system usage.

In conclusion, the aforementioned studies of this research topic, accompanied by numerous other investigations, provide valuable insights into the acceptance, benefit evaluation, and cost implications associated with digital health services. As the ongoing proliferation of innovations persistently reconfigures global healthcare infrastructures, it is indispensable to eradicate prospective obstacles and facilitate egalitarian accessibility to superior care, thereby engendering unprecedented possibilities through the utilization of, and access to, data derived from digital health applications among other resources. Collaborative efforts among stakeholders, including policymakers, healthcare professionals, and developers, will be vital in overcoming these barriers (8).
